# Investigating antibiotic resistance in non-clinical environments

**DOI:** 10.3389/fmicb.2013.00019

**Published:** 2013-02-15

**Authors:** Fiona Walsh

**Affiliations:** Department of Bacteriology, Federal Department of Economic Affairs, Forschungsanstalt Agroscope Changins-WädenswilWädenswil, Switzerland

**Keywords:** susceptibility testing, soil, definition of resistance, resistome, guidelines

## Abstract

There have been many calls for more information about the natural resistome and these have also highlighted the importance of understanding the soil resistome in the preservation of antibiotics for the treatment of infections. However, to date there have been few studies which have investigated the culturable soil resistome, which highlights the difficulties faced by microbiologists in designing these experiments to produce meaningful data. The World Health Organization definition of resistance is the most fitting to non-clinical environmental studies: antimicrobial resistance is resistance of a microorganism to an antimicrobial medicine to which it was previously sensitive. The ideal investigation of non-clinical environments for antibiotic resistance of clinical relevance would be using standardized guidelines and breakpoints. This review outlines different definitions and methodologies used to understand antibiotic resistance and suggests how this can be performed outside of the clinical environment.

The World Health Organization World Health Day 2011 highlighted the problems of antibiotic resistance under the title “Antibiotic resistance: no action today, no cure tomorrow”. Every year 25,000 people in the European Union die because of a serious resistant bacterial infection, mostly acquired in health care settings^1^. The search for antibiotics, understanding their mechanisms of action and the development and spread of antibiotic resistance has resulted in the development of many industries and novel research areas for over 100 years. However, the number of antibiotics coming to the market over the past 30 years has dramatically declined as many pharmaceutical companies and biotech firms abandoned the search for antibiotics in favor of other pharmaceuticals (Spellberg et al., 2004). Antibiotic resistance has developed over time from resistance to single classes of antibiotics to multi-drug resistance and extreme drug resistance. Until recently, antibiotics and antibiotic resistance were thought of in terms of treatments of infections and the prevention of successful treatment, respectively. The mechanisms of action and resistance have been studied almost exclusively in pathogenic bacteria. It is only in recent years that research in antibiotic resistance has focused on the environment from which the antibiotics were initially extracted: soil microorganisms and the soil ecosystem. With an every decreasing supply of novel antibiotics and increasing resistance the emphasis has turned to defining the natural antibiotic resistome and understanding the ecology and evolution of antibiotic resistance in the non-clinical environment. These recent research focuses are thought to bring answers to help prevent a return to the pre-antibiotic era.

There have been many calls for more information about the natural resistome and these have also highlighted the importance of understanding the soil resistome in the preservation of antibiotics for the treatment of infections (Pruden et al., 2006; American Academy of Microbiology, 2009; Aminov, 2009; Rosenblatt-Farrell, 2009). However, to date there have been few studies which have investigated the culturable soil resistome, in antibiotic producing bacteria *Streptomyces*, and an isolated cave microbiome (D’Costa et al., 2006; Bhullar et al., 2012). Numerous studies have been performed using polymerase chain reaction (PCR) or quantitative PCR (qPCR) to screen the environment for known resistance genes or more recently metagenomics (Volkmann et al., 2004; Chen et al., 2007; Knapp et al., 2008; Lata et al., 2009; Zhang et al., 2011; McGarvey et al., 2012; Popowska et al., 2012). Functional metagenomics have also been used to identify novel resistance mechanisms in soil (Allen et al., 2009; Donato et al., 2010; Torres-Cortés et al., 2011). The identification of antibiotic resistance hotspots and the understanding of the evolution and ecology of antibiotic resistance in the environment are novel areas of research. There have been more reviews written on this topic that research papers to date, which highlights the difficulties faced by microbiologists in designing these experiments to produce meaningful data. We are faced with some fundamental difficulties in assessing and analyzing the environmental antibiotic resistome.

Antibiotic resistance and antibiotic breakpoints have been defined within the context of their medical functions. Clinical breakpoints define bacteria as susceptible, intermediate, or resistant to an antibiotic and are calculated using several factors, including clinical results from studies, wild type minimum inhibitory concentration (MIC) distributions for the relevant bacterial species, antibiotic dosing and pharmacokinetic (PK) and pharmacodynamics (PD) measurements (Clinical and Laboratory Standards Institute [CLSI], 2006; European Committee on Antimicrobial Susceptibility Testing [EUCAST], 2011). This is used as a guide for the clinician to decide how to treat the patient, with antibiotic resistance meaning treatment failure. But what does antibiotic resistance mean out-with the human health or infection treatment context? We need to also define what an antibiotic is in the natural environment, as this too is a term adapted for use in terms of treating bacterial infections.

The soil may be a reservoir of resistance genes, which are already present in human pathogens or which may emerge to increase the current arsenal of antibiotic resistance mechanisms in pathogens. Most antibiotics used in human medicine have been isolated from soil microorganisms. Therefore, soil is thought of as a potential reservoir of antibiotic resistance genes. The presence of antibiotics in soil is believed to have promoted the development of highly specific antibiotic resistance mechanisms in antibiotic producing and non-producing bacteria (D’Costa et al., 2006). This belief is based on studies, which have identified resistance genes such as *bla*_CTX-M_, *qnrA*, and *bla*_NDM_ as originating in the environmental bacteria *Kluyvera* spp., *Shewanella algae*, and *Erythrobacter litoralis*, respectively (Oliver et al., 2001; Nordmann and Poirel, 2005; Zheng et al., 2011). These genes are clinically relevant resistance genes and are currently causing difficulties in the treatment of bacterial infections. The origins of other plasmid mediated resistance genes are still unknown. Anthropocentrism has led to the view that genes such as *bla*_CTX-M_, *qnrA*, and *bla*_NDM_ have evolved in nature as antibiotic resistance genes. However, the true functions of these genes remain to be characterized. By approaching antibiotic resistance in nature not from an anthropocentric viewpoint, but from bacterial evolution and ecological standpoints will we be able to identify the origins and evolution of such genes.

While soil may be a reservoir of antibiotic resistance genes, but not all resistance mechanisms are necessarily a threat to the continued use of antibiotics in all pathogens. Intrinsic resistance is a characteristic of almost all isolates of the bacterial species (Leclercq et al., 2011). Intrinsic resistance occurs when the antimicrobial activity of the drug is clinically insufficient or antimicrobial resistance is innate, rendering it clinically ineffective (Leclercq et al., 2011). The commonly believed theory of the role of the soil resistome is that antibiotic production and resistance co-exist in soil bacteria, as demonstrated by studies of antibiotic biosynthetic pathways and genome analysis (Cases and de Lorenzo, 2005; D’Costa et al., 2007). The theory is that without the resistance gene the antibiotic producing bacteria would self-destruct, on production of the antibiotic. However, Davies and Davies (2010) pointed out that this theory remains to be proven. In order to understand the importance of soil as a potential reservoir of antibiotic resistance mechanisms we need to investigate these theories. The soil may be a reservoir of antibiotic resistance genes, but we need to ask which resistance mechanisms are relevant to clinical antibiotic use?

The human pathogen is not the ancestral home of antibiotic resistance as they have developed to infect humans, not to live in soil with antibiotic producers. Therefore, if soil is the natural reservoir of antibiotic resistance the most important resistance mechanisms are those that can transfer from soil bacteria to pathogenic bacteria. Thus, we need to study soil bacteria as a reservoir of antibiotic resistance with relevance to the clinical use of antibiotics. Another reason to study the soil resistome is to understand the roles of antibiotics and resistance in nature: the natural ecology and evolution of resistance. The idea is that by learning more about how resistance has developed over time we can understand antibiotic resistance evolution and spread within patients and also help to predict the future evolution of resistance to existing and novel antibiotics. Our use of antibiotics has changed the course of antibiotic resistance ecology and evolution. The environment does not exist in a separate world to that of humans. There is a constant flow to and from soil, especially in urban and agricultural environments. Human activities such as using antibiotics in the treatment of human and animal diseases or in agriculture, but also pollution and climate change have altered the soil environment. If the soil is a reservoir of antibiotic resistance mechanisms, we need to identify how our actions and climatic change will also affect the soil resistome. The topic of ecology and evolution of resistance in the environment will be discussed in other papers within this special topic review and will not be addressed in this review.

We frequently refer to bacteria as being resistant to antibiotics, but rarely do we consider what that means. Even the most resistant bacterium can be inhibited or killed by a sufficiently high concentration of antibiotic; patients, however, would not be able to tolerate the high concentration required in some cases (Hawkey, 1998). In order to study the antibiotic resistome, we need to know what antibiotic and antibiotic resistance means in terms of soil bacteria. Antibiotic action and resistance up until a few years ago has been studied almost exclusively in terms of human or animal pathogens. We can separate the study of the soil antibiotic resistome into two different contexts:

1. Clinical relevance: antibiotic resistance of relevance to pathogens
2. Natural relevance: ecology and evolution of antibiotic resistance

These separations ensure that when we study antibiotic resistance in clinical terms that we have a definition of antibiotics and antibiotic resistance, as they are used in medicine. I will focus on the clinical relevance for the remainder of this review. The term antibiotic was described by Waksman (1973) as a description of a use, a laboratory effect or an activity of a chemical compound. Davies and Davies (2010) defined an antibiotic as any class of organic molecule that inhibits or kills microbes by specific interactions with bacterial targets, without any consideration of the source of the particular compound or class. Antibiotic resistance from a clinical viewpoint has been defined by the European Agency for the evaluation of medicinal products, as microbiologically resistant or clinically resistant^2^.

Microbiological resistance:

“Resistant microorganisms from a microbiological point of view are those that possess any kind of resistance mechanism or resistance gene.” This definition is quantified using MIC data and breakpoints for the antibiotics.

Clinical resistance:

“The classification of a bacteria as susceptible or resistant depends on whether an infection with the bacterium responds to therapy.”

Clinical resistance is a complex concept in which the type of infecting bacterium, its location in the body, the distribution of the antibiotic in the body and its concentration at the site of infection, and the immune status of the patient all interact. The difficulty arises when we try to apply these definitions to soil bacteria or non-pathogenic bacteria, where no breakpoints exist. If we identify a novel resistance mechanism in a soil bacteria, will this resistance gene cause resistance in a human pathogen? The antibiotic resistance definition of most relevance in both clinical and non-clinical environments is that of the WHO:

Antimicrobial resistance is resistance of a microorganism to an antimicrobial medicine to which it was previously sensitive.

In order to define antibiotic resistance in non-clinical environments we need to address the specific context and define what is meant by sensitive. In terms of clinically relevant antibiotic resistances, we can define resistance in all contexts as bacteria containing a known resistance gene or those, which are no longer inhibited at the site of infection. These bacteria would then be considered resistant to the respective antibiotics. Defining an antibiotic sensitive bacteria is a more complex task, especially with respect to bacteria inhabiting different environments. A bacteria defined as sensitive may be so in soil, but in the presence of clinically relevant antibiotic concentrations, may be highly mutable or have inducible resistance mechanisms. Thus, a sensitive bacteria is one, which would not be readily selected in the presence of higher concentrations of antibiotic than those concentrations in the environment. If an environmental species in a particular place increases its MIC to a certain antibiotic along a limited period of time it can be considered that it has become “more” resistant or less susceptible. However, defining resistance by the presence of known resistance genes is limiting the search for resistance to those already characterized. Thus, we propose that such resistance genes be separated into pR-genes, potential resistance genes or pre-resistance genes and aR-genes, genes known to produce an antibiotic resistance phenotype in bacteria capable of survival and integration into the human or animal microbiotia.

How do we decide which resistances are relevant? In clinical practice we know that *Pseudomonas aeruginosa* is intrinsically resistant to ampicillin due to chromosomally mediated AmpC, efflux and impermeability. Therefore, clinical *Pseudomonas aeruginosa* are not tested for susceptibility to ampicillin as it is not used to treat *Pseudomonas* *aeruginosa* infections. However, if soil is described as a potential reservoir of resistance genes, which can move from the chromosome to mobile elements and then to pathogens we need to test all of the bacteria, not just the pathogens or antibiotic producers. If we identify a penicillin resistant *Pedobacter *species, does this mean that *Pedobacter* contains a potential novel resistance gene or, like *Pseudomonas* *aeruginosa* it is mediated by intrinsic resistance or that it is only or importance if it can be expressed in pathogenic bacteria?

Novel resistance genes may be identified using functional metagenomics; total soil DNA is extracted, digested, and ligated into a vector. The vector is transferred into a bacterial host, e.g., *Escherichia coli* and the functional characteristics are measured using clinical breakpoints (Handelsman, 2004). However, in hospitals antibiotic resistance is generally tested using MIC or disk diffusion assays. A definition of antibiotic resistance for antibiotic susceptibility testing of bacteria in non-clinical environments is required. The culture-based studies of antibiotic resistance in soil bacteria to date have defined antibiotic resistance as growth at 20 mg/L (D’Costa et al., 2007; Bhullar et al., 2012). This arbitrary definition is based on the use of 20 mg/L as the breakpoint concentration in the initial soil resistome study of *Streptomyces* species (D’Costa et al., 2007). This definition is used for all bacteria and all classes of antibiotics.

The clinical breakpoint of an antibiotic is determined by combining the relevant factors in setting breakpoints for antimicrobial agents and consist of, as defined by the European Committee on Antimicrobial Susceptibility Testing:

1. Available formulations
2. Standard and maximum dosing
3. Clinical indications and target organisms
4. MIC distributions for individual species
5. Pharmacokinetic (PK) data in humans
6. Pharmacodynamic (PD) data
7. Information from modeling processes
8. Clinical data relating outcome to MIC values
9. Information on resistance mechanisms, the clinical significance of the resistance mechanisms, and the MICs for organisms expressing the resistance mechanisms

However, where no PK/PD data for antibiotics with a particular species have been generated the breakpoints should be based on epidemiological cut-off (ECOFF) values for the antibiotics^3^. In the case of clinical infections these are limited to antibiotics used to treat the infection. In a recent evaluation of *Pasteurella multocida* by EUCAST the ECOFF values were determined using approximately 250 isolates for the tests and were estimated by visual inspection or statistically calculated (Turnidge et al., 2006). Ideally greater than 250 isolates would be obtained from multiple centers or countries in order to establish a worldwide ECOFF values. Each country could then survey their own multi-center sites in order to identify edaphic influences on the soil resistome. Although the culturable bacteria represent less than 1% of the total bacterial population, within this 1% remains a large number of bacterial species, for which there are no antibiotic breakpoint values. The possibility of culturing many more organisms than this 1% will be provided by the advances in “culturomics” and combining metagenomics data with concurrent sequencing (Lagier et al., 2012; VanInsberghe et al., 2013). In order to define a bacteria as resistant we need to test the MIC distribution within the population and identify the breakpoint. Therefore, in terms of defining antibiotic breakpoints for bacteria from non-clinical environments the ECOFF would be the most appropriate. In order to create breakpoints for these environments we would need to collect and test at least 250 isolates from different locations, in order to have a representative sample. With breakpoints we can set standard guidelines for susceptibility testing of antibiotics in non-clinical environments. Susceptibility testing is the gold standard of antibiotic resistance testing used throughout the world in hospitals. It is a relatively cheap and easy technique with little need for sophisticated or expensive equipment. Therefore, using susceptibility testing would enable the comparison of non-clinical data with clinical data. However, this is limited to culturable bacteria.

Non-culture-based techniques are required to investigate the entire bacterial community. The bacteria that can be grown in the laboratory are only a small fraction of the total diversity that exists in nature. Approximately only 1% of bacteria on Earth can be readily cultivated *in vitro* (Staley and Konopka, 1985; Amann et al., 2001). Therefore, non-culture-based tools such as PCR and metagenomics are required to capture the non-culturable section of the non-clinical antibiotic resistome. However, one disadvantage of these tools are that they are limited to screening for known resistance genes and mechanisms and identification of only the resistance gene rather than being able to investigate the process involved in resistance, as with culturable bacteria. PCR and quantitative PCR have been frequently used to determine the presence of resistance genes in nature and the effects of agriculture on the emergence and spread of resistance. The most frequently used methods to determine the presence of antibiotic resistance in the environment have been PCR detection, microarray detection or real-time PCR detection of known resistance genes (Chen et al., 2007; Koike et al., 2007; Peak et al., 2007; Walsh and Rogers, 2008; Borjesson et al., 2009; Walsh et al., 2010). These techniques are limited to detecting genes, which are known and have been sequenced. More recently functional metagenomics have identified novel resistance genes and antibiotic biosynthesis genes present in environmental bacteria (Donato et al., 2010; Torres-Cortés et al., 2011). The direct applications of sequencing have to date been mostly focused on the elucidation of species variations within the environment (Kohler et al., 2005; Janssen, 2006; Lauber et al., 2009; Shange et al., 2012).

The application of functional metagenomics on soil DNA have identified novel resistance genes and antibiotic biosynthesis genes present in environmental bacteria (Donato et al., 2010; Torres-Cortés et al., 2011). Functional metagenomics is the genomic study, without culturing, of a population of microorganisms (Handelsman, 2004). This approach has been applied to the study of the antibiotic resistome present in environmental samples (Riesenfeld et al., 2004; Donato et al., 2010; Torres-Cortés et al., 2011). The advantages of metagenomics are that the bacterial community can be analyzed for antibiotic resistance genes without the need to culture these organisms and can be used to detect as yet unknown antibiotic resistance genes. The gene’s function is expressed as the antibiotic resistance phenotype in the host bacteria. However, the disadvantage lies with the fact that the host bacteria is frequently* Escherichia coli*, which may not be capable of expressing all of the resistance genes, e.g., *strA*.

Antibiotic resistance genes and plasmids have been identified in pristine and agricultural ecosystems using PCR, functional metagenomics and pyrosequencing tools (Pruden et al., 2006; Allen et al., 2009; Heuer and Smalla, 2012). Both novel resistance genes and bifunctional genes were identified in soil from apple orchards (Donato et al., 2010). The revolution in high-throughput sequencing has brought unique opportunities and challenges to the field of environmental microbiology. Since its introduction in 2005, the number of metagenomic libraries in the database has increased year by year and following the quantum leaps of next generation sequencing techniques (Schmieder and Edwards, 2012). Dependent on the sequencing platform, up to 500 Gb of sequencing data can be generated per single run (Shokralla et al., 2012).

The complete characterization of the soil antibiotic resistome requires both culture and non-culture-based approaches. Using defined guidelines and definitions the scientific community can standardize the methodologies required. This has proved very effective in the surveillance of antibiotic resistance, emerging trends, and novel resistance mechanisms in clinical bacteria. The advantage of identifying the bacteria associated with the resistance mechanism, if it is a novel mechanism, is that the entire genetic cascade or pathway leading to resistance can be studied in depth. However, culture-based techniques alone will only describe a small fraction of the bacteria present in non-clinical environments. A combination of culture-based and non-culture-based standardized techniques will provide a wealth of information on the composition of the non-clinical antibiotic resistome. The WHO definition of resistance is the most fitting to non-clinical environmental studies. The ideal investigation of non-clinical environments for antibiotic resistance of clinical relevance would be using standardized guidelines and breakpoints as outlined in this review.

## Conflict of Interest Statement

The author declares that the research was conducted in the absence of any commercial or financial relationships that could be construed as a potential conflict of interest.

## Footnotes

1 http://www.who.int/world-health-day/2011/en/

2 http://www.ema.europa.eu/docs/en_GB/document_library/Report/2009/10/WC500005166.pdf

3 http://www.eucast.org/
